# Probiotic Properties of *Enterococcus* Isolated From Artisanal Dairy Products

**DOI:** 10.3389/fmicb.2019.00300

**Published:** 2019-02-26

**Authors:** Yousef Nami, Reza Vaseghi Bakhshayesh, Hossein Mohammadzadeh Jalaly, Hajie Lotfi, Solat Eslami, Mohammad Amin Hejazi

**Affiliations:** ^1^Department of Food Biotechnology, Branch for Northwest and West Region, Agricultural Biotechnology Research Institute, Education and Extension Organization (AREEO), Tabriz, Iran; ^2^Dietary Supplements and Probiotic Research Center, Alborz University of Medical Sciences, Karaj, Iran

**Keywords:** *Enterococcus*, probiotic properties, dairy products, low cholesterol, antimicrobial activity, safety evaluation, Enterococcus as probiotics, virulence factors

## Abstract

The present study focused on probiotic characterization and safety evaluation of *Enterococcus* isolates from different artisanal dairy products. All the isolates exhibited inhibitory activity against several food spoilage bacteria and food-borne pathogens, including *Shigella flexneri, Staphylococcus aureus, Listeria monocytogenes, Yersinia enterocolitica, Klebsiella pneumoniae, Escherichia coli*, and *Bacillus subtilis*. The PCR results indicated the presence of at least one enterocin structural gene in all the tested strains. The *Enterococcus* isolates were further evaluated regarding their safety properties and functional features. The isolates were susceptible to vancomycin, gentamycin, and chloramphenicol. The results of PCR amplification revealed that all the tested isolates harbored none of the tested virulence genes except *E. faecalis* (ES9), which showed the presence of *esp* gene. The *Enterococcus* isolates showed cholesterol lowering properties. The selected isolates showed a high tolerance to low pH, and toward bile salts. They also demonstrated hydrophobicity activity, auto-aggregation, and adhesion ability to the human intestinal Caco-2 cell line. These properties may contribute the bacteria colonizing the gut. This study revealed that the *Enterococcus* isolates, especially *E. durans* ES11, ES20 and ES32, might be excellent candidates for production of functional foods to promote health benefits.

## Introduction

Enterococci are belonging to genera of lactic acid bacteria (LAB). They are Gram-positive, catalase negative, cocci-shaped, facultative anaerobe, and non-spore forming bacteria (Haghshenas et al., 2016). Based on phylogenetic evidence and molecular studies (16S-rDNA sequencing or DNA–DNA hybridization), more than 26 species were classified in this genus. These microorganisms are ubiquitous bacteria which present as common microbiota in the intestine of humans, mammals, and other animals gastrointestinal tracts, but they are also exist in soil, water, vegetable products, meats, fermented and cooked meat and dairy products (Li et al., 2018; Zommiti et al., 2018). This is due to their high tolerance to harsh conditions such as high temperatures, low pH and high salinity. Significant role of *E. faecium, E. faecalis*, and *E. durans* in the ripening of traditional cheeses have indicated that enterococci play an important role in the ripening of these cheeses, probably through proteolysis, lipolysis, and citrate breakdown, hence contributing to their typical taste and flavor. A majority of works specify that *Enterococcus* isolates play a vital role in the development of the sensory properties of fermented foods like olives (they break down oleuropein in fermented olives), sausages and cheese (Moreno et al., 2006).

There are different arrays of probiotics, mostly *Lactobacillus* and *Bifidobacteria* groups and *Enterococcus* genera in recent years, are used in functional foods (Haghshenas et al., 2017). The claimed advantageous of probiotic enterococci are: (i) diarrhea or diarrhea treatment in association with antibiotic medication, viral contaminations, chemotherapy and diseases originated from food-borne pathogens (Lau and Chamberlain, 2016); (ii) curbing the pathogenic bacteria growth (Zorriehzahra et al., 2016); (iii) anti-mutagenic and anti-carcinogenic features; (iv); increased intestinal mucosal barrier (Ahl et al., 2016); (v) stimulation of the immune system (Sheikhi et al., 2016); (vi) prevention of ulcers related to *Helicobacter pylori* infection (Oh et al., 2016) and (vii) cholesterol assimilation in food and human intestine (Kobyliak et al., 2016). These microorganisms have antagonistic activities through pathogens by different antimicrobial compounds production comprises bacteriocins, lactic and acetic acids and hydrogen peroxide.

However, due to association of some enterococci with human infections, like urinary tract infections, bloodstream infections, bacteraemia, endocarditis and diarrhea and surgical site infections; concerns about the safety of these bacteria have raised the attention of health organizations to use as probiotic bacteria because their virulence aspects contribute in human infections (Brandão et al., 2010; Zommiti et al., 2018). Generally, vancomycin-resistant enterococci (VRE) in nosocomial infections is considered as a major problem (Arias and Murray, 2012). Furthermore, the action of many virulence genes have been elucidated in *Enterococcus* isolates (Carlos et al., 2010). The most important virulence factors are *cyl*A, *cyl*B and *cyl*M, *esp, agg, gel*E, *cpd, ccf*, and *cad* genes. The gene *cyl*A is responsible for the cytosilin transportation and activation. The genes *cyl*B and *cyl*M have an application in modification of post-translational, while a cell wall protein concerned in the immune evasion is associated to *esp* gene. Adherence to eukaryotic cells is associated to an aggregation protein which is encoded by *agg* gene. *gel*E is responsible for the production of toxin which hydrolyzes gelatin, and finally sex pheromones which are responsible for facilitating conjugation are encoded by *cpd, ccf*, and *cad* genes (Belgacem et al., 2010; Liu et al., 2015). The aim of this study was to isolate and identify *Enterococcus* isolates from traditional dairy products, evaluation of their safety, probiotic aptitudes, and antimicrobial properties due to high potential roles of enterococci in health and food.

## Materials and Methods

### Sampling and Culture Conditions

Artisanal dairy products (yogurt, cheese, and curd) were collected from domestic producers (Table 1). The samples were transported to the laboratory in ice boxes and stored at 4°C. For better separation of bacteria from solid particles of yogurt and cheese, initial homogenization took place by vortexing. To prepare the bacterial suspension of yogurt, 10 g of yogurt was transferred to 100 mL of sterile physiological peptone water and shaken gently. To prepare the bacterial suspension of cheese and curd, 20 g of each sample were suspended in 180 mL of tri-sodium citrate sterile solution, and after half an hour, 10 mL of prepared solution were added to 200 mL de Man Rogosa Sharpe (MRS) broth in order to enrich and enhance the initial bacterial population in anaerobic conditions and incubated at 37°C for 24 h (Haghshenas et al., 2017).

**Table 1:** Origin, region of samples prepared, and acid and bile tolerance of isolates.

Isolates	Origin	Region	Survival rate (%) at pH 2.5	Survival rate (%) at 0.3% bile
ES1	Cheese	Sarab	25.1o	26.6m
ES2	Curd	Ahar	17.1s	16.9t
ES3	Yogurt	Kaleybar	29.2j	30.2j
ES4	Cheese	Sarab	69.7d^**^	70.1d^**^
ES5	Yogurt	Heris	33.2i	31.1i
ES6	Yogurt	Heris	15.5u	14.1w
ES7	Curd	Ahar	12.6v	9.1z
ES8	Yogurt	Kaleybar	34.3h	36.6f
ES9	Cheese	Kaleybar	66.2f	69.1de
ES10	Curd	Ahar	26.4m	26.2m
ES11	Cheese	Heris	76.3c^**^	79.8a^**^
ES12	Curd	Kaleybar	37.3g	34.1g
ES13	Yogurt	Sarab	25.7n	27.1kl
ES14	Yogurt	Heris	21.2q	22.1o
ES15	Cheese	Sarab	18.2r	19.1q
ES16	Yogurt	Sarab	10.3x	11.2y
ES17	Yogurt	Kaleybar	17.2s	18.2r
ES18	Yogurt	Kaleybar	28.1k	27.2kl
ES19	Yogurt	Kaleybar	15.9t	15.5u
ES20	Cheese	Heris	81.6a^**^	79.1ab^**^
ES21	Curd	Sarab	25.3o	33.1h
ES22	Yogurt	Heris	27.6l	24.2n
ES23	Yogurt	Sarab	11.3w	12.3x
ES24	Cheese	Kaleybar	25.2o	26.4m
ES25	Curd	Ahar	15.3v	14.9v
ES26	Yogurt	Heris	22.2p	21.4p
ES27	Yogurt	Sarab	68.4e^**^	68.3e^**^
ES28	Yogurt	Sarab	68.1c^**^	74.3c^**^
ES29	Curd	Ahar	18.1s	17.7s
ES30	Curd	Ahar	25m	26.1m
ES31	Cheese	Heris	26.3kl	27.1kl
ES32	Yogurt	Heris	77.4b^**^	78.2b^**^


*^*a–z*^Means in the same color with different lowercase letters differed significantly (*p* < 0.05). ^∗∗^Means are significantly different (*p* < 0.01).*

### Isolation of *Enterococcus* Isolates

*Enterococcus* isolates were isolated by the streak-plate method on MRS agar and incubated aerobically at 37°C for 24 h. The single colonies were routinely checked for purity by microscopic examination. The pure colonies were used to characterize Gram staining and catalase test. The colonies which were Gram-positive and catalase-negative were selected and inoculated in MRS broth containing 30% glycerol as cryo-protectant and stored at -80°C (El Soda et al., 2003). The purified cultures were activated by sub-culturing twice in MRS broth before use.

### Assessment of Probiotic Properties

#### Acid and Bile Salts Tolerance

To determine acid tolerance, 10 mL of bacterial culture of each sample were incubated for 24 h in MRS broth. Selected colonies were transferred into mineral medium phosphate-buffered saline (PBS, pH 2.5). The samples were incubated aerobically for 3 h at 37°C. Afterward, the cells were diluted up to 10 times using sterile saline (sodium chloride: 5.8 g/L) and each dilution of 100 μL for MRS agar surface in culture medium was cultured. The samples were incubated aerobically for 48–72 h at 37°C (Haghshenas et al., 2015).

Tolerance to bile salts was analyzed based on the method used previously by Nami et al. (2015b). Briefly, MRS broth culture medium, as a control, and MRS with 0.3% bile oxgall, used as a test medium (treatments), were inoculated simultaneously with 1% of active bacterial culture at 37°C for 4 h. Optical densities of the control and treated cultures growth were measured by a spectrophotometer (Eppendorf, Germany) at 600 nm. The percentage of growth suppression was measured by using the following formula:

$$\%\;of\;suppresion=\frac{Growth\;in\;Control\;broth-Growth\;in\;bile\;broth}{Growth\;in\;control\;broth}\times 100$$

#### Antimicrobial Activity and Bacteriocin Detection

Well diffusion method was performed to conclude and recognize the inhibitory metabolites produced by *Enterococcus* isolates (Nami et al., 2015a). Overnight cultures of the selected isolates were cultured in MRS agar at 37°C for 24 h. Indicator bacteria used in this study were *Shigella flexneri* PTCC 1234, *Staphylococcus aureus* ATCC 25923, *Listeria monocytogenes* ATCC 13932, *Yersinia enterocolitica* ATCC 23715, *Klebsiella pneumoniae* PTCC 1053, *Escherichia coli* PTCC, 1276 and *Bacillus subtilis* ATCC 19652. These pathogenic organisms were purchased from the Persian Type Culture Collection (PTCC) to detect the antagonistic substances. Half McFarland indicator bacteria (1.5 × 10^8^ CFU/mL) were poured on Mueller-Hinton agar and the wells were cut on plates. Then, each well was filled by 50 μL of filtered supernatant and plates incubated overnight at 37°C and finally, the inhibition zone around the wells was measured by digital calipers.

The proteinaceous nature of the inhibition was assessed. To this end, the active cell-free culture supernatants were obtained by centrifugation at 15000 RPM for 12 min at 4°C. They were subjected to various enzyme treatments, including catalase, trypsin, α-chymotrypsin, and proteinase K, at 1 mg/mL at 37°C for 2 h, after adjusting the pH at 6.2 with 1 M of NaOH. Then, the residual activity was assessed against pathogenic microorganisms. The protease sensitivity was determined by the absence of inhibition zones around the wells. To confirm the presence of hydrogen peroxide, the active supernatants were subjected to sterilized catalase (1 mg/mL) and incubated at 37°C for 2 h and finally their activities were assessed by the well diffusion method.

#### PCR Amplification of Known Enterocin Genes

All the structural genes concerned to the expression of well-known enterocins *EntA, EntB, EntP, EntL50A, EntL50B, Ent31* (Toit et al., 2000), *EntQ* (Belgacem et al., 2010), and *Ent1071* (Omar et al., 2004) were amplified with specific PCR primers (Table 2). PCR amplification was performed at a final volume of 50 μL that comprised of 1 Taq polymerase buffer, 200 μM of dNTP’s, 25 pM of each primer, 2 μL of template DNA (stock) and 1 U of Taq DNA polymerase (Thermo Fisher Scientific, United States). The PCR products were visualized by electrophoresis on 2% agarose gels.

**Table 2:** Primers used for PCR amplification of virulence factors and enterocin detection genes in Enterococcus strains.

Gene^∗^	Sequence (5′-3′)	Ta (°C)	Amplicon size (bp)	Reference	Enterocin^∗∗^	Sequence (5′-3′)	Reference
cylA	F: ACTCGGGGATTGATAGGC R: GCTGCTAAAGCTGCGCTT	54	688	Creti et al., 2004	EntA	F: AAATATTATGGAAATGGAGTGTAT R: GCACTTCCCTGGAATTGCTC	Toit et al., 2000
clyB	F: ATTCCTACCTATGTTCTGTTA R: AATAAACTCTTCTTTTCCAAC	56	843	Vankerckhoven et al., 2004	EntB	F: GAAAATGATCACAGAATGCCTA R: GTTGCATTTAGAGTATACATTTG	Toit et al., 2000
esp	F: AGATTTCATCTTTGATTCTTGG R: AATTGATTCTTTAGCATCTGG	56	510	Vankerckhoven et al., 2004	EntP	F: TATGGTAATGGTGTTTATTGTAAT R: ATGTCCCATACCTGCCAAAC	Toit et al., 2000
gelE	F: ACCCCGTATCATTGGTTT R: ACGCATTGCTTTTCCATC	56	402	Mannu et al., 2003	EntQ	F: ATGAATTTTCTTCTTAAAAATGGTATCGCA R: TTAACAAGAAATTTTTTCCCATGGCAA	Belgacem et al., 2010
asa1	F: GCACGCTATTACGAACTATGA R: TAAGAAAGAACATCACCACGA	56	375	Vankerckhoven et al., 2004	EntL50A	F: TGGGAGCAATCGCAAAATTAG R: ATTGCCCATCCTTCTCCAAT	Toit et al., 2000
Ace	F: GAATTGAGCAAAAGTTCAATCG R: GTCTGTCTTTTCACTTGTTTC	56	320	Mannu et al., 2003	EntL50B	F: TGGGAGCAATCGCAAAATTAG R: ATTGCCCATCCTTCTCCAAT	Toit et al., 2000
efaAfs	F: GACAGACCCTCACGAATA R: AGTTCATCATGCTGTAGTA	56	705	Eaton and Gasson, 2001	Ent1071	F: CCTATTGGGGGAGAGTCGGT R: ATACATTCTTCCACTTATTTTT	Omar et al., 2004
cpd	F: TGGTGGGTTATTTTTCAATTC R: TACGGCTCTGGCTTACTA	50	782	Eaton and Gasson, 2001	Bac31	F: TATTACGGAAATGGTTTATATTGT R: TCTAGGAGCCCAAGGGCC	Toit et al., 2000


*Ta (°C), Annealing temperature; bp, base pairs.*

#### Exopolysaccharide (EPS) Production

The method used by Fguiri et al. (2016) was used for assessment of EPS production ability of isolates. Briefly, the cultures were streaked on m-MRS agar medium which was modified by replacing glucose with 100 g/L of sucrose and incubated at 37°C for 24 h aerobically. Metal loop was used to drag up formed colonies. If the length of slime was above 1.5 mm, the isolate was considered positive slimy producers.

#### Cell Surface Hydrophobicity

The adhesion ability of isolates to xylene was determined as previously described by Mishra and Prasad (2005).

#### Auto-Aggregation and Co-Aggregation

The ability of the isolates to auto-aggregate was performed according to the method described by Angmo et al. (2016). Auto-aggregation percentage was determined using the following equation:

1−(At/A0)×100

Where A0 represents absorbance at t = 0 and at represents absorbance at time t.

Co-aggregation of *Enterococcus* isolates against the seven pathogens was performed at 37°C after 4 h of incubation based on method used by Zuo et al. (2016). Co-aggregation percentage was calculated based on equation:

$$\%=\frac{A0-\mathrm{At}}{\mathrm{At}}\times 100$$

#### Adhesion Ability to Human Intestinal Cells

Adhesion ability to human colon carcinoma cells (Caco-2) was evaluated as reported previously by Nami et al. (2014). Briefly, the Roswell Park Memorial Institute (RPMI-1640; Sigma) medium, supplemented with 10% heat-inactivated fetal bovine serum, was used to culture the human cells. The cells were cultured on 24-well tissue culture plates and incubated at 37°C in 5% CO_2_ in a relatively humid atmosphere until a confluent monolayer was achieved. The viable Caco-2 cells were counted in a Burker haematocytometer chamber. Then, the cell suspension including bacteria and Caco-2 cells was subjected to pure plate technique to determine C.F.U. bacteria adhesion was expressed as the total number of bacteria attached to viable Caco-2 cells.

#### Cholesterol Assimilation

Cholesterol removal percentage was determined by o-phthalaldehyde method described by Rudel and Morris (1973) with some alteration. A freshly prepared MRS broth was supplemented with 0.3% oxgall (Merk Germany) as bile salt and water-soluble cholesterol (150 μg/mL) was added as the cholesterol source (sterilized by 0.2 μL filter), the mixture inoculated with each isolate at 1% level and incubated anaerobically at 37°C for 20 h. The cells were removed by centrifugation (10000 rpm for 15 min) after the incubation period; subsequently, 1 mL of the cell-free broth was mixed with 1 mL KOH (33% W/V) and 2 mL ethanol 96%, vortexed for 2 min, followed by heating at 60°C for 15 min. Mixes cooled in room temperature, 2 mL distilled water and 3 mL hexane were added and vortexed for 1 min. One mL the hexane layer was transferred into a glass tube and evaporated in water bath at 80°C. The residue was immediately dissolved in 2 mL *o*-phthalaldehyde (Merck, Germany) reagent, Followed by 0.5 mL concentrated sulphuric acid and vortexed completely for 1 min. The samples were incubated at room temperature for 30 min and finally absorbance was read at 550 nm.

#### β-Galactosidase Activity

β-galactosidase activity of *Enterococcus* isolates was assessed according to Angmo et al. (2016). Bacterial cultures were streaked on MRS agar plates containing 60 μL X-gal (5-bromo-4-chloro-3-indolyl-β-D-galactopyranoside) and 10 μL of IPTG (iso-propyl-thio-β-D-galactopyranoside) solution as inducer. The presence of β-galactosidase activity in strains was determined after 42 h of incubation at 37°C.

### Safety Assessment

#### Hemolytic Activity

Haemolytic activity of *Enterococcus* isolates was examined by culturing of fresh overnight cultures on Columbia agar plates (Oxoid) containing 7% (v/v) sheep blood (Oxoid) and incubated for 48 h at 37°C. Finally, hemolytic activities were detected by 3 categories: appearance of a halo around the colony: greenish zone considered as *α*-hemolysis, clear zone for *β*-hemolysis and no halo for γ-haemolysis (Abedi et al., 2018).

#### Bile Salts Hydrolysis

The bile salt hydrolysis was evaluated according to Argyri et al. (2013). The hydrolysis activity was indicated by 0.5% (w/v) taurodeoxycholic acid after 48 h of incubation at 37°C. The hydrolysis activity was evaluated by the formation of opaque agar halos around the colonies.

#### Detection of Virulence Factors

PCR amplification was performed to detect genes encoding potential virulence factors. Total bacterial DNA was isolated by the method described by Leenhouts et al. (1990). Virulence genes evaluated in this study were *cylA, clyB, esp, gelE, asa1, Ace, efaAfs*, and *cpd*. *E. faecium* ATCC 51299 was used as a control. PCR amplification was performed to detect genes encoding these factors using several primers (Table 2). The PCR amplification was carried out in 0.2 mL reaction tubes each with 25 μL of mixtures using 0.1 mM of deoxynucleoside triphosphates, 2.5 mM of MgCl_2_, 0.5 mM of each primer, PCR Buffer (1X), 2 U of Taq polymerase and 50 ng/μL of DNA template. PCR amplifications were performed with a cycle of initial denaturation (94°C for 5 min), followed by 32 cycles of denaturation (94°C for 60 s), annealing at an appropriate temperature (Table 2) for 60 s and elongation (72°C for 5 min). The PCR products were analyzed by gel electrophoresis in 1.5% agarose stained with ethidium bromide (0.5 g/mL).

#### Antibiotic Susceptibility

Antibiotic susceptibility test was carried out based on previously described method of Haghshenas et al. (2014). Briefly, disk diffusion assay was performed to determine antibiotic sensitivity of the isolates. MRS agar medium was used for this test. Antimicrobial disks (5 mm) were purchased from Padtan Teb Co, Iran. The tested antibiotics were vancomycin (30 μg), colistin (10 μg), streptomycin (10 μg), cefepime (30 μg), cefixime (15 μg), sulfamethoxazole (2 μg), kanamycin (30 μg), ciprofloxacin (5 μg), tetracycline (30 μg), erythromycin (15 μg), ampicillin (10 μg), gentamycin (10 μg), clindamycin (30 μg), ceftriaxon (30 μg), chloramphenicol (30 μg), and cefalexin (30 μg), using disk diffusion method. After overnight incubation at 37°C, the diameter of inhibition (mm) around each disk was measured.

### 16S-rDNA Gene Sequencing

Amplification of 16S-rDNA gene (1500 bp) of the isolate was performed by using a pair of lactic acid bacteria (LAB)-specific universal primers (Hal6F/Hal6R) (F: 5′-AGAGTTTGATCMTGGCTCAG-3′ and R: 5′-TACCTTGTTAGGACTTCACC-3′) previously described by Haghshenas et al. (2016). The PCR amplification was fulfilled for the total volume 50 μL under the following conditions: initial denaturation at 94°C for 5 min, followed by 35 cycles of denaturation at 94°C for 45 s, annealing at 59°C for 60 s, extension at 72°C for 90 s, and a final extension step at 72°C for 10 min. The PCR products were visualized through 1% (w/v) agarose gel (Sigma Chemical Co., Poole, United Kingdom) electrophoresis and stained via ethidium bromide. The PCR products were sent to the Macrogen DNA Sequencing Service (Korea) to be sequenced. Multiple sequence alignment of 16S rRNA genes was carried out using CLUSTAL W function (Thompson et al., 1994) with default parameters, and a phylogenetic tree of 16S rRNA genes was reconstructed using the neighbor-joining method (Saitou and Nei, 1987) implemented in the MEGA X software (Kumar et al., 2018) with p-distance parameter distance. Bootstrap values were calculated with 1,000 re-samples. The 16S rRNA gene sequence of *Lactobacillus acidophilus* (LC064893.1) was used as out-group for the analysis.

### Statistical Analysis

Statistical analysis of data was carried out using SPSS (Ver. 19.0 SPSS, Chicago, IL, United States). The comparisons of differences between the means of the treatments were analyzed by one-way ANOVA at a significance level of *P* < 0.05. All the experiments were performed in triplicate and data expressed as means ± standard deviations.

## Results

### Characterization and Identification of Isolates

From 90 isolates from various dairy products of different regions of East Azerbaijan Province in Iran, 66 resulted isolates were gram-positive, catalase-negative and rod- or cocci-shaped bacteria. Among them, 32 isolates were cocci-shaped and exhibited optimum growth at 37°C but not at 30°C (data not shown). From 32 cocci-shaped isolates, 8 resulted isolates were from cheese, 8 from curd, and 16 from yogurt (Table 1). Based on morphological and biochemical assays, the authors assumed that these isolates are likely to be *Enterococcus* strains.

### Acid and Bile Salt Tolerance

A stimulated *in vitro* gastric juice (pH 2.5) was used to assess the acid tolerance profile of *Enterococcus* isolates (Table 1). The survival rate of isolates displayed a significant variability, ranging from 10.3 to 81.6%. The highest survival rate was observed for isolate ES20 (81.6 ± 0.3%), followed by isolates ES32 (77.4 ± 0.3%) and ES11 (76.3 ± 0.2%), while the lowest survival rate was observed for isolate ES16 with 10.3 ± 0.1% survival rate.

The percentage of viability of *Enterococcus* isolates was assessed after 4 h of incubation in M-17 broth supplemented with 0.3% oxgall (Table 1). The viability rate of isolates exhibited a significant variability ranging from 09.1 ± 0.1 to 79.8 ± 0.3%. The highest viability rate belonged to isolate ES11 (79.8 ± 0.3%), followed by isolates ES20 (79.1 ± 0.2%) and ES32 (78.2 ± 0.3%), while the lowest viability rate belonged to isolate ES7 (09.1 ± 0.1%). Among the 32 isolates, only isolates 4, 9, 11, 20, 27, 28, and 32 exhibited more than 50% acid and bile tolerance. Hence, these seven isolates were selected for further investigation.

### Antimicrobial Activity and Detection of Enterocin Genes

The inhibitory effect of isolated *Enterococcus* isolates against some important pathogenic microorganisms is shown in Table 3. The results showed that all the seven selected isolates were capable of inhibiting the growth of the majority of target pathogens. The target pathogens used in this study were *Shigella flexneri, Staphylococcus aureus, Listeria monocytogenes, Yersinia enterocolitica, Klebsiella pneumoniae, Escherichia coli*, and *Bacillus subtilis* (Table 4). Isolates ES20 and ES28 were able to inhibit the growth of all the tested pathogens. Moreover, isolates ES9, ES11, ES27, and ES32 were able to inhibit the growth of all the target pathogens except *Shigella flexneri.*

**Table 3:** The inhibitory effect of selected *Enterococcus* strains against pathogenic microorganisms.

Isolates	Indicator pathogens							Detection of enterocin structural genes							
		
	*Escherichia coli*	*Shigella flexneri*	*Klebsiella pneumoniae*	*Yersinia enterocolitica*	*Listeria monocytogenes*	*Bacillus subtilis*	*Staphylococcus aureus*	EntA	EntB	EntP	EntQ	Ent L50A	Ent L50B	Ent 1071	Bac 31
ES28	34.3 ± 0.2	22 ± 0.3	8.4 ± 0.1	8.9 ± 0.2	12.9 ± 0.2	35.9 ± 0.2	33.2 ± 0.2	+	+	+	-	-	-	-	-
ES11	30.2 ± 0.1	–	13.1 ± 0.2	13.1 ± 0.3	21.6 ± 0.3	23.2 ± 0.2	17.5 ± 0.3	+	+	-	+	+	-	-	-
ES32	20.3 ± 0.1	–	13.1 ± 0.3	33.3 ± 0.3	27.6 ± 0.3	13.4 ± 0.2	21.9 ± 0.3	+	+	-	+	+	-	-	-
ES27	9.1 ± 0.2	–	8.2 ± 0.2	13.2 ± 0.3	11.2 ± 0.2	8.7 ± 0.2	7.9 ± 0.2	+	+	+	-	-	-	-	-
ES4	–	7.3 ± 0.3	9.8 ± 0.3	–	22.3 ± 0.2	9.3 ± 0.3	15.9 ± 0.2	+	+	+	-	-	-	-	-
ES20	25 ± 0.1	32.4 ± 0.6	18.1 ± 0.3	17.3 ± 0.2	22.2 ± 0.2	22.1 ± 0.3	26.1 ± 0.2	+	+	-	+	+	-	-	-
ES9	22.3 ± 0.2	–	17.3 ± 0.2	18.5 ± 0.3	8.4 ± 0.2	13.2 ± 0.2	22.2 ± 0.2	+	+	-	-	-	-	-	-


*Values are mean ± standard error of triplicates. (Strong ≥ 20 mm), (Moderate < 20 mm > 10 mm), and (Weak ≤ 10 mm).*

**Table 4:** The origin of indicator pathogenic bacteria used in this study.

Indicator pathogens	Culture Collection	Code
*Shigella flexneri*	Persian Type Culture Collection (PTCC)	1234
*Staphylococcus aureus*	American Type Culture Collection (ATCC)	25923
*Listeria monocytogenes*	American Type Culture Collection (ATCC)	13932
*Yersinia enterocolitica*	American Type Culture Collection (ATCC)	23715
*Klebsiella pneumoniae*	Persian Type Culture Collection (PTCC)	1053
*Escherichia coli*	Persian Type Culture Collection (PTCC)	1276
*Bacillus subtilis*	American Type Culture Collection (ATCC)	19652


When pH adjusted to 6.2, the isolates ES4, ES9, ES11, and ES27 could not inhibit the growth of any pathogens. Also, the isolates ES20 and ES32 do not able to inhibit the growth of *Shigella flexneri, Listeria monocytogenes, Klebsiella pneumoniae*, and *Bacillus subtilis.* So, it resulted that the nature of inhibition of these isolates are because of acid production. Furthermore, after subjecting other isolates to catalase enzyme, none isolates were able to inhibit the growth of indicator pathogens, except isolates ES28 and ES32 against *Staphylococcus aureus* and *Yersinia enterocolitica*, respectively. To confirm the nature of inhibition of isolate ES28 against *Staphylococcus aureus*, and isolate ES32 against *Yersinia enterocolitica*, protease K enzyme was subjected. After applying this enzyme, the clear zone of inhibition was removed and it showed that the nature of inhibition is because of bacteriocin production.

Extracted DNA of *Enterococcus* isolates was subjected to PCR amplification to determine the existence of structural genes coding *EntA, EntB, EntP, EntL50A, EntL50B*, and *Ent31* enterocins (Table 3). The PCR results indicated the presence of at least one enterocin structural genes in all the 7 isolates. The enterocins A and B structural genes were detected among all the isolates and the enterocins P, Q and L50A were found in 3 isolates. On the other hand, none of the evaluated isolates showed PCR amplification fragments for other tested enterocins (entL50B, ent1071, and bac31).

### Cell Surface Hydrophobicity

**FIGURE 1:**
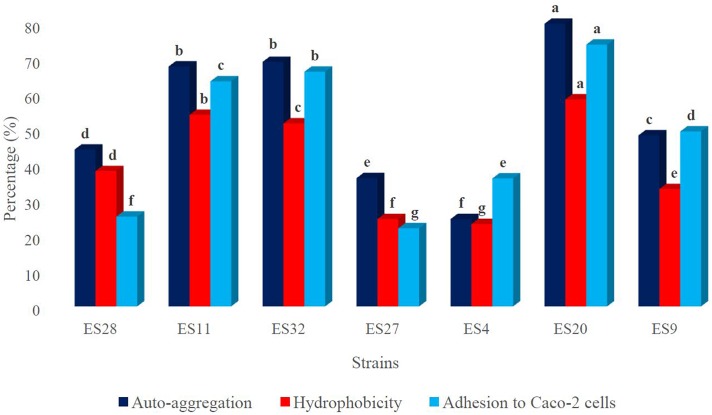
The hydrophobicity, auto-aggregation and adhesion ability of strains to human intestinal cells. ^a-g^Means in the same color with different lowercase letters differed significantly (*p < 0.05*).

The cell surface hydrophobicity rate is illustrated in Figure 1. It ranged from 23.3 ± 1.6 to 58.6 ± 2.3%. The highest cell hydrophobicity rate was observed for isolates ES20, followed by ES11 and ES32 with 58.6 ± 2.3%, 54.2 ± 1.9% and 51.8 ± 1.4, respectively. Furthermore, isolates ES4 and ES27 showed the lowest cell hydrophobicity rates with 23.3 ± 1.6 and 24.7 ± 1.3%, respectively.

### Adhesion Capacity to Intestinal Cells

The adhesion capacity to human colon carcinoma cell line, Caco-2, was determined (Figure 1). Adhesion capacity to Caco-2 cells varied significantly among the tested bacteria, with adhesion ratio ranging from 22.1 ± 1.8 to 74.1 ± 1.9%. The highest adherence capacity belonged to isolates ES20, ES32, and ES11, with mean values of 74.1 ± 1.9, 66.4 ± 2.2, and 63.7 ± 1.8%, respectively.

### EPS Production Ability

The ability of isolates to produce EPS is illustrated in Table 5. The results demonstrated that all the isolates exhibited EPS production ability.

**Table 5:** Origin, Molecular identification, average cholesterol-removal ratio, BSH activity, EPS production, hemolytic activity and β-galactosidase activity of strains after 20 h of growth at 37°C.

Strain	Molecular identification	Accession number	BSH activity^∗^	Assimilated cholesterol rate (μg/mL)	Cholesterol assimilation rate (%)	Haemolytic activity^∗∗^	β-galactosidase activity	EPS production^∗∗∗^
ES28	*E. hirae* ABRIINW.N2	MK367693	-	99.93	33.31	-	-	+
ES11	*E. durans* ABRIINW.N3	MK367694	+++	172.23	57.41	-	+	+
ES32	*E. durans* ABRIINW.F	JQ366081.1	+++	175.38	58.46	-	+	+
ES27	*E. faecium* ABRIINW.M	JQ366083.1	++	144.42	48.14	-	+	+
ES4	*E. faecium* ABRIINW.N	JQ366084.1	+	145.23	48.41	-	-	+
ES20	*E. durans* ABRIINW.N1	MK367581	+++	216.45	72.15	-	+	+
ES9	*E. faecalis* ABRIINW.L	JQ366082.1	-	123.63	41.21	-	-	+


*^∗^BSH activity was expressed based on the diameters of precipitation zones: **–**, no precipitation; +, >10 mm; ++, >15 mm; and +++, >20 mm. ^∗∗^ (-) no haemolysis; (α and β) haemolysis. ^∗∗∗^ (–) no EPS production; (+) EPS production.*

### Auto-aggregation and Co-aggregation

The results of cell auto-aggregation assay are shown in Figure 1. The cell auto-aggregation rates of the isolates ranged from 24.7 ± 2.3 to 81.2 ± 2.6%. The highest scores were obtained for isolates ES20, followed by ES32, and ES11 with 81.2 ± 2.6, 69.2 ± 2.1, and 67.9 ± 1.2%, respectively. Furthermore, isolate ES4 showed the lowest auto-aggregation rates with 24.7 ± 2.3%.

The results of co-aggregation of *Enterococcus* isolates in the presence of *Staphylococcus aureus, Escherichia coli, Listeria monocytogenes, Shigella flexneri, Klebsiella pneumoniae, Yersinia enterocolitica*, and *Bacillus subtilis* separately at 37°C at 2 and 4 h of incubation are shown in Table 6. The results showed that isolates 11, 20, and 32 exhibited higher co-aggregation ability compared to other isolates. The co-aggregation percentages increased (*P < 0.05*) during incubation. Co-aggregations of *Enterococcus* isolates with all the pathogens at 4 h of incubation were higher (*P < 0.05*) compared to 2 h of incubation. Isolates demonstrated lower co-aggregation (*P < 0.05*) toward gram-positive pathogens (*S. aureus, L. monocytogenes*, and *B. subtilis*) compared to gram-negative ones (*Y. enterocolitica, Sh. Flexneri, K. pneumoniae*, and *Escherichia coli*).

**Table 6:** Co-aggregation (%) of *Enterococcus* isolates with 7 pathogens during 4 h incubation at 37°C.

Isolates	Time (hour)	*Pathogenic bacteria*						
		
		*S. aureus*	*E. coli*	*L. monocytogenes*	*Sh. flexneri*	*K. pneumoniae*	*Y. enterocolitica*	*B. subtilis*
ES11	2	0.9 ± 1.7^b^	10.8 ± 0.97^b^	11.4 ± 1.18^c^	8.1 ± 1.16^d^	12.3 ± 1.22^d^	13.3 ± 1.98^c^	8.1 ± 1.08^d^
ES20		6.8 ± 1.25^c^	10.9 ± 1.94^b^	8.8 ± 1.47^d^	11.8 ± 1.67^c^	11.9 ± 0.98^d^	12.3 ± 0.48^c^	10.7 ± 1.26^c^
ES27		1.7 ± 0.32^fg^	2.7 ± 0.83^g^	1.9 ± 0.51^h^	2.1 ± 0.44^f^	2.3 ± 0.67^g^	2.5 ± 0.36^f^	2 ± 0.67^h^
ES28		1.2 ± 0.93^g^	3.5 ± 0.91f^g^	1.9 ± 0.94^h^	6.4 ± 1.27^d^	7.3 ± 1.53^e^	9.37 ± 1.20^d^	1.7 ± 0.64^hi^
ES32		6.7 ± 1.4^c^	10.8 ± 0.9^b^	7.3 ± 1.2^de^	11.4 ± 1.29^c^	14.5 ± 1.94^c^	12.5 ± 1.25^c^	7.9 ± 1.32^d^
ES4		3.7 ± 0.46^def^	5.2 ± 1.34^ef^	3.9 ± 0.97^fg^	4.2 ± 0.81^ef^	4.4 ± 0.58^fg^	4.7 ± 0.68^ef^	4 ± 1.04^fg^
ES9		3.7 ± 0.86^def^	6.2 ± 1.1^de^	4.3 ± 0.84^fg^	6.4 ± 0.88^d^	5.9 ± 0.66^ef^	6.7 ± 0.75^e^	4.7 ± 0.95^ef^
ES11	4	12.7 ± 1.3^a^	19.9 ± 1.42^a^	18.7 ± 1.47^a^	14.8 ± 1.23^b^	16.7 ± 1.41^b^	19.6 ± 1.95^b^	13.9 ± 1.77^ab^
ES20		10.7 ± 1.8^b^	19.5 ± 1.57^a^	13.8 ± 1.24^b^	21.4 ± 1.58^a^	22.6 ± 1.34^a^	23.1 ± 2.2^a^	14.7 ± 1.09^a^
ES27		2.2 ± 0.25^efg^	2.8 ± 0.24^g^	2.5 ± 0.34^gh^	3.4 ± 0.25^f^	3.1 ± 0.42^g^	2.9 ± 0.38^f^	2.4 ± 0.61^gh^
ES28		4.2 ± 1.65^de^	8.5 ± 1.7^c^	5.8 ± 1.41^ef^	10.7 ± 1.64^c^	12.1 ± 1.82^d^	12.7 ± 1.69^c^	6.4 ± 1.59^de^
ES32		9.9 ± 1.15^b^	17.8 ± 1.2^a^	11.13 ± 1.4^c^	19.7 ± 1.67^a^	20.9 ± 1.58^a^	19.3 ± 1.38^b^	12.4 ± 1.19^bc^
ES4		4.1 ± 0.66^de^	5.9 ± 1.29^de^	4.3 ± 0.27^fg^	6.1 ± 0.98^de^	5.7 ± 0.88^ef^	5.9 ± 0.62^e^	4.4 ± 0.67^f^
ES9		5.2 ± 0.81^cd^	7.9 ± 0.82^cd^	5 ± 0.64^f^	7.7 ± 0.82^d^	6.9 ± 1.07^e^	9.1 ± 0.82^d^	5.7 ± 0.47^ef^


*Values are mean ± standard error of triplicates. ^*a*-*i*^Means in the same column with different lowercase letters differed significantly (*p < 0.05*).*

### Cholesterol Assimilation and Bile Salt Hydrolysis

Table 5 presents the levels of cholesterol assimilation by isolates in the presence of 0.3% bile oxgall at 37°C for 20 h. The content of cholesterol removed varied (*P* < 0.05) and ranged from 99.93 to 216.45 μg/mL. The highest content of cholesterol assimilation was observed in isolates ES32, ES20, and ES11, which belonged to *Enterococcus durans* species with 216.45, 175.38, and 172.23 μg/mL. In contrast, the lowest content belonged to isolate ES28.

The bile salt hydrolysis of the *Enterococcus* isolates is shown in Table 5. The results indicated that isolates ES11, ES20, and ES32 showed the highest BSH activity (+++), whereas isolate ES27 exhibited moderate BSH activity (++). Furthermore, isolate ES4 showed less BSH activity (+), while isolates ES9 and ES28 showed no activity (-).

### Hemolytic Activity

Hemolytic activity of isolates is represented in Table 5. All the isolates showed no β-hemolytic activity.

### β-Galactosidase Activity

Isolates ES11, ES20, ES27, and ES32 indicated the presence of β-galactosidase activity, while isolates ES4, ES7, and ES28 did not show the presence of this enzyme.

### Detection of Virulence Factors

The presence of genes encoding eight known virulence factors in the *Enterococcus* isolates was assessed. The results of PCR amplification revealed that none of the isolates harbored any virulence factors except *E. faecalis* (ES9), which showed the presence of *esp* gene.

### Antibiotic Susceptibility

Table 7 illustrates the antibiotic resistance of *Enterococcus* isolates against 16 tested antibiotics. Overall, all the isolates showed the ability to resist the impact of tetracycline and colistin, whilst all the isolates were susceptible to gentamycin, vancomycin and chloramphenicol. The impact of other antibiotics against isolates varied from susceptible to resistant. Among the tested *Enterococcus* isolates, isolate ES11 was susceptible to all the antibiotics, except for tetracycline and colistin.

**Table 7:** Antibiotic susceptibility of strains.

Strains	Antibiotic susceptibility)zone of inhibition in mm)													
	
	V	CL	S	FEB	CFM	SXT	K	CP	TE	E	AM	GM	CC	CRO	C	CN
ES28	S	R	S	S	I	S	I	R	R	I	R	S	S	S	S	S
ES11	S	R	S	S	S	S	S	I	R	S	S	S	S	S	S	S
ES32	S	R	S	S	S	S	S	I	R	S	S	S	S	S	S	S
ES27	S	R	R	R	S	S	S	R	R	S	S	S	R	S	S	R
ES4	S	R	R	S	R	R	S	R	R	S	R	S	R	I	S	R
ES20	S	R	S	S	S	S	S	S	R	S	S	S	S	S	S	S
ES9	S	R	R	R	S	S	I	R	R	I	R	S	R	R	S	R


*V, vancomycin; CL, colistin; S, streptomycin; FEB, cefepime; CFM, cefixime; SXT, sulfamethoxazole; K, kanamycin; CP, ciprofloxacin; TE, tetracycline; E, erythromycin; AM, ampicillin; GM, gentamycin; CC, clindamycin; CRO, ceftriaxon; C, chloramphenicol; CN, cefalexin and CL, colistin. Erythromycin results based on R ≤ 13 mm; I: 13–23 mm; S ≥ 23 mm. Gentamycin results based on R ≤ 6 mm; I: 7–9 mm; S ≥ 10 mm. Vancomycin results based on R ≤ 12 mm; I: 12–13 mm; S ≥ 13 mm. I: intermediate (zone diameter, 12.5–17.4 mm); R: resistant (zone diameter, ≤12.4 mm); S: susceptible (zone diameter, ≥17.5).*

### 16S-rDNA Sequencing

**FIGURE 2:**
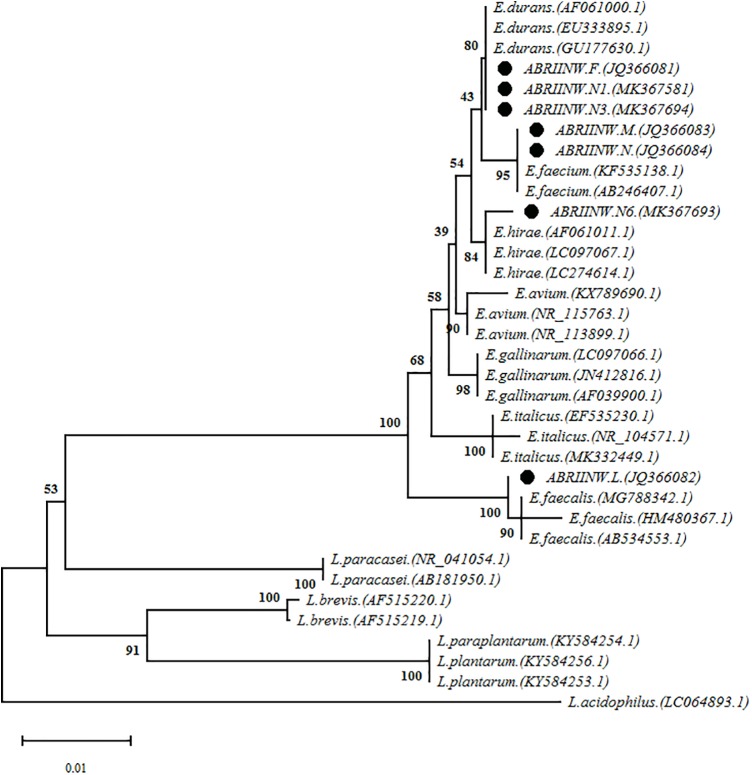
The analysis of the phylogeny of the isolated *Enterococcus* strains alignment of the sequences was performed with the sequences of different *Enterococcus* and *Lactobacillus* species which were submitted in NCBI database as complete sequence.

16S-rDNA sequencing was performed as molecular phylogeny analysis to identify selected *Enterococcus* isolates at the species level. Phylogenetic tree (Figure 2) was constructed based on the 16S-rDNA sequences from evolutionary distances by the neighbor-joining method. Analysis of the sequences depicted that isolate ES9 clustered with sequences of *Enterococcus faecalis*, isolates ES4 and ES27 clustered with sequences of *Enterococcus faecium*, isolate ES28 clustered with *Enterococcus hirae* and three isolates ES11, ES20 and ES32 clustered with *Enterococcus durans*.

## Discussion

The ability of isolates to survive under high acidic conditions and to show acceptable tolerance against bile salts in the human intestine are two key properties for a candidate to be considered a probiotic (Kandylis et al., 2016; Ayyash et al., 2018). In this study, the survival rate of isolates in acidic conditions and bile salts displayed a significant variability, which might be due to the fact that mechanisms of acid and bile tolerance are species and strain-dependent. Isolates ES4, ES9, ES11, ES20, ES27, ES28, and ES32 showed favorable acid and bile tolerance compared to the other isolates. Therefore, only these seven isolates were subjected to further tests. The acid and bile tolerances of the seven isolates are consistent with the results reported by Nami et al. (2014); El-Jeni et al. (2015), Haghshenas et al. (2016), and Ayyash et al. (2018).

The nature of inhibitory effect of isolates was assessed by adjusting pH to 6.2 and also using catalase and protease enzymes. After treating with protease enzymes, the clear zones around the halos were disappeared. It could be because of proteinaceous nature of secreted metabolites by isolates. It has been shown by some studies (Balla et al., 2000; Cintas et al., 2000) that bacteriocins secreted by *Enterococcus* isolates are strong inhibitors of food-borne pathogens such as *S. aureus, L. monocytogenes* and *Clostridium tyrobutyricum*. In our study, the inhibitory profile of the *Enterococcus* isolates under assessment tended to be active against a wide range of gram-positive and gram-negative bacteria and food-borne pathogens, including *Staphylococcus, Listeria, Yersinia, Bacillus, Shigella, Escherichia coli* and *Klebsiella*. These recorded activities are in contrast with Stevens et al. (1991) and Zommiti et al. (2018), who theorized that bacteriocins of LAB are ineffective against gram-negative bacteria because the outer membrane blocks the bacteriocin target. Moreover, PCR amplification of genes coding for enterocins (*EntA, EntB, EntQ, EntP, EntL50A, EntL50B, Ent1071*, and *Bac31*) was investigated. All the isolates contained at least one enterocin gene and the enterocins A and B were detected in all the strains. This is consistent with the results reported by Cintas et al. (2000) and Belgacem et al. (2010), who detected these putative enterocin factors in *Enterococcus* isolates. Isolates ES11, ES20, and ES32, which showed the ability to compete against all the seven tested pathogens, contained a combination of four enterocins such as entA, entB, entQ and entL50A. This is consistent with Sánchez et al. (2007), who proposed the co-production of two or more enterocins by a strain generating supernatants with a higher antagonistic activity.

Probiotic capacity to remain alive in the gastrointestinal tract is one of the most desirable features of probiotics. To be colonized in the intestine, probiotics have to adhere to the intestinal mucosa to avoid being removed from the colon by peristalsis. In this study, strains ES20, ES32, and ES11 exhibited favorable adherence capacity (Duary et al., 2011; Kumar et al., 2015). Similar to these results, high capability to adhere to Caco-2 cells was reported for *Enterococcus* isolates (Cebrián et al., 2012; Pimentel et al., 2012).

The ability of isolates to produce EPS was determined by the presence of ropy white mucus on skimmed milk plates containing ruthenium red. It has been shown that LAB is able to produce EPS, which improves the viscosity and texture of dairy products. Hence, EPS-producing LAB is widely used in the dairy industry. The presence of (glyco-) proteinaceous on the cell surface results in higher hydrophobicity, while the presence of polysaccharides leads to hydrophilic surfaces (Osmanagaoglu et al., 2010).

Hydrophobicity is one of the indicative parameters for cell surface properties of probiotics, which correlates with the adhesion ability of probiotics to epithelial cells (Duary et al., 2011; Zuo et al., 2016; Ayyash et al., 2018). Thus, the higher hydrophobicity resulted in higher ability of probiotics to attach to epithelial cells and promote health benefits. In the current study, isolates ES20, ES11, and ES32 exhibited better hydrophobicity percentages compared with the results reported by Ayyash et al. (2018). Moreover, Das et al. (2016) also reported that hydrophobicity of three LAB ranged from 22.2 to 25.0%, which is lower as compared with our findings.

The auto-aggregation and co-aggregation ability are two important properties of probiotics, which are defined as the bacterial accumulation of the same species and of different species, respectively (Campana et al., 2017). The auto-aggregation and co-aggregation are fundamental for probiotics because it seems that auto-aggregation is correlated with adherence to epithelial cells (Collado et al., 2008), while co-aggregation represents a defensive barrier for the colonization of pathogenic microorganisms (Kos et al., 2003; Abushelaibi et al., 2017). In addition, the bacterial equilibrium in the gastrointestinal tract is increased by aggregation of probiotics in the human gut (Tulumoglu et al., 2013) and the probiotic properties of the LAB are improved by their co-aggregation ability in the presence of gut pathogens. The formation of a defensive barrier because of co-aggregation of LAB in the presence of pathogens will not allow pathogens to colonize in the human gut (Vidhyasagar and Jeevaratnam, 2013). Tareb et al. (2013) reported that the ability of LAB isolates to co-aggregate with pathogens could be attributed to proteinaceous components present on the cell surface and interactions between carbohydrate and lectin. Nevertheless, Collado et al. (2008) revealed that the co-aggregation ability of LAB is time- and strain- dependent. Our results correspond with those of Angmo et al. (2016); Taheur et al. (2016), and Abushelaibi et al. (2017). Our study showed that the co-aggregation ability is significantly affected by incubation time and strain.

*In vitro* studies on cholesterol reduction by *Enterococcus* species have been considered as an important parameter for the selection of probiotic strains with diverse health-promoting benefits. The hypocholesterolemic effect on host is another important but not essential property of probiotics. Several mechanisms have been postulated for lowering cholesterol by probiotic bacteria (Miremadi et al., 2014), including conversion of cholesterol to coprostanol by reductase, cholesterol incorporation in the cell wall and disruption of cholesterol micelle in the intestine by deconjugated bile salts. Cholesterol removal results in the current study are in agreement with the results of studies by Ayyash et al. (2018). In the current study, the cholesterol removal could be attributed mainly to cholesterol micelle in the intestine by deconjugated bile salts.

Presently the role of BSH is controversial because it might act either positively in lowering of serum cholesterol or negatively in increasing the level of undesirable deconjugated bile salts (Xie et al., 2015; Fadda et al., 2017). On the other hand, BSH activity by probiotic bacteria might be desirable because it increases the intestinal survival and persistence of producing strains, which in turn increases the overall beneficial effects associated with the strain (Begley et al., 2006; Park et al., 2007).

The safety assessment is obligatory before a strain is qualified as beneficial for the host health for use in food industry. The absence of hemolytic activity and antibiotic resistance are the basic requirements for the selection of safe probiotic strains (Oh and Jung, 2015). No β-hemolytic activity was detected in the 7 tested strains.

The presence of β-galactosidase, which is a useful enzyme that hydrolyses lactose into glucose and galactose was performed. Lactose intolerance is due to the lack or shortage of this enzyme; hence lactose mal-digestion symptoms could be improved by consumption of probiotics that release β-galactosidase (Hussain et al., 2008). On the other hand, the products fermented with β-galactosidase producers play an essential role in the treatment of lactose intolerance (Vasiljevic and Jelen, 2001). Our findings proved that the tested enterococci isolates showed good β-galactosidase activity. Isolates ES32, ES20 and ES11 showed the highest β-galactosidase activity. Hence, these isolates may have an effective application in the dairy industry and also for the treatment of lactose intolerance.

A desirable characteristic for enterococcal bacteria used in food industry is the absence of cytolysin-encoding genes. Cytolysin is a bacterial toxin expressed by some *E. faecalis* isolates. In our study, only three isolates showed the presence of cytolysin-encoding genes, which belonged to *E. faecalis* isolates. None of the isolates belonging to *E. durans, E. hirae* and *E. faecium* showed the presence of these genes. In addition, only five isolates belonging to *E. faecalis* showed the presence of *esp* gene. The absence of *esp* in *E. faecium* was reported (Eaton and Gasson, 2001), which revealed the frequent presence of the esp gene in medical *E. faecium* isolates. Our results are in accordance with the results of Eaton and Gasson (2001) and Liu et al. (2015). Overall, the presence of virulence genes is higher in *E. faecalis* species than in *E. faecium* species, which is consistent with our results.

The resistance of *Enterococcus* species to various antibiotics has been reported by some studies (Vidhyasagar and Jeevaratnam, 2013). Because of transferring the resistance factors from probiotics to pathogenic microorganisms via the interchange of genetic materials, the intake of antibiotic-resistant strains disrupts the original flora in the intestine (Mathur and Singh, 2005; Hummel et al., 2007; Taheur et al., 2016). In the current study, all the isolates were resistant to colistin and tetracycline. This can be attributed to the overuse of these antibiotics in rural areas.

## Conclusion

The results of this study indicated that *E. durans* ES11, *E. durans* ES20 and *E. durans* ES32 are safe probiotic strains with the potential to assimilate total cholesterol. These strains fulfilled several criteria to be used as probiotic microorganisms, including auto- and co-aggregation ability, resistance to low pH and high bile salts, adherence to hydrocarbons, susceptibility to some antibiotics as well as EPS production. As these isolates are from the food sources which display a wide spectrum of capability against certain intestinal and food-borne pathogens, it could be used in functional foods since these probiotic strains adapt to the conditions and could provide protection against pathogens. Further studies will be required to determine the mechanisms underlying the cholesterol-lowering effect and to evaluate the long-term probiotic potential of these strains.

## Author Contributions

All authors listed have made a substantial, direct and intellectual contribution to the work, and approved it for publication.

## Conflict of Interest Statement

The authors declare that the research was conducted in the absence of any commercial or financial relationships that could be construed as a potential conflict of interest.
